# Supplementary material for: Effects of Pueraria candollei var mirifica (Airy Shaw and Suvat.) Niyomdham on Ovariectomy-Induced Cognitive Impairment and Oxidative Stress in the Mouse Brain
Source: Molecules. 2021 Jun 5;26(11):3442. doi: 10.3390/molecules26113442 (PMC8201258; doi:10.3390/molecules26113442)
Supplement: Supplementary file 1 [file molecules-26-03442-s001.zip › molecules-1224184-supplementary.pdf]

Supplementary Materials

Effects of *Pueraria candollei* var *mirifica* (Airy Shaw and Suvat.) Niyomdham on Ovariectomy-Induced Cognitive Impairment and Oxidative Stress in the Mouse Brain

Yaowared Chulikhit [1], Wichitsak Sukhano [1], Supawadee Daodee [1], Waraporn Putalun [2], Rakvajee Wongpradit [1], Charinya Khamphukdee [2], Kaoru Umehara [3,4], Hiroshi Noguchi [3,5], Kinzo Matsumoto [6] and Orawan Monthakantirat [1,*]

[1] Division of Pharmaceutical Chemistry, Faculty of Pharmaceutical Sciences, Khon Kaen University, Khon Kaen 40002, Thailand; yaosum@kku.ac.th (Y.C.); Berzerkwiz@gmail.com (W.S.); csupawad@kku.ac.th (S.D.); rakvajeewpd@kkumail.com (R.W.)
[2] Division of Pharmacognosy and Toxicology, Faculty of Pharmaceutical Sciences, Khon Kaen University, Khon Kaen 40002, Thailand; waraporn@kku.ac.th (W.P.); charkh@kku.ac.th (C.K.)
[3] Department of Pharmacognosy, School of Pharmaceutical Sciences, University of Shizuoka, Yada 52-1, Shizuoka-shi, Shizuoka 422-8526, Japan; kaoru.umehara@hamayaku.ac.jp (K.U.); noguchi@u-shizuoka-ken.ac.jp (H.N.)
[4] Faculty of Pharmaceutical Sciences, Yokohama University of Pharmacy, Yokohama, Kanagawa 245-0066, Japan;
[5] Department of Pharmacognosy, Nihon Pharmaceutical University, Saitama, 362-0806, Japan
[6] Division of Medicinal Pharmacology, Institute of Natural Medicine, University of Toyama, 2630 Sugitani, Toyama 930-0194, Japan; mkinzo@inm.u-toyama.ac.jp
* Correspondence: oramon@kku.ac.th; Tel.: +664-320-2305

---

1. *Effects of PM extract and estrogen on OVX-induced cognitive impairments*

Table S1. One-way analysis of variance (ANOVA) test of Y-maze test

Group comparison	ANOVA followed by Tukey' s post hoc test	
	P	F ($DF_{between\ group}$, $DF_{residual}$)
Sham VS. OVX Vehicle-treated	<0.001	$F(3,39)=16.376$
OVX VS. OVX+E_2 (1 μg/kg/day)	<0.001	
OVX VS. OVX+PM2.5 (2.5 mg/kg/day)	<0.05	
OVX VS. OVX+PM25 (25 mg/kg/day)	<0.001	

Table S2. One–way analysis of variance (ANOVA) test of novel objective recognition test (NORT)

Group comparison	ANOVA followed by Tukey's post hoc test	
	P	F ($DF_{between\ group}$, $DF_{residual}$)
Sham VS. OVX Vehicle–treated	<0.001	F(3,42) =19.890
OVX VS. OVX+E_2 (1 μg/kg/day)	<0.001	
OVX VS. OVX+PM2.5 (2.5 mg/kg/day)	Not significant	
OVX VS. OVX+PM25 (25 mg/kg/day)	<0.001	
OVX+PM2.5 VS. OVX+PM25	<0.001	

Table S3. One-way analysis of variance (ANOVA) test of the morris water maze task

Group comparison Training test (Day 2)	ANOVA followed by Tukey's post hoc test	
	P	F ($DF_{between\ group}$, $DF_{residual}$)
Sham VS. OVX Vehicle–treated	<0.05	F(4,53) =13.405
OVX VS. OVX+E_2 (1 μg/kg/day)	<0.05	
OVX VS. OVX+PM2.5 (2.5 mg/kg/day)	Not significant	
OVX VS. OVX+PM25 (25 mg/kg/day)	<0.05	
**Group comparison Training test (Day 3)**	**ANOVA followed by Tukey's post hoc test**	
	P	F ($DF_{between\ group}$, $DF_{residual}$)
Sham VS. OVX Vehicle–treated	<0.001	F(4,53) =9.192
OVX VS. OVX+E_2 (1 μg/kg/day)	<0.05	
OVX VS. OVX+PM2.5 (2.5 mg/kg/day)	<0.001	
OVX VS. OVX+PM25 (25 mg/kg/day)	<0.001	
**Group comparison Training test (Day 4)**	**ANOVA followed by Tukey's post hoc test**	
	P	F ($DF_{between\ group}$, $DF_{residual}$)
Sham VS. OVX Vehicle–treated	<0.05	F(4,49) =12.004
OVX VS. OVX+E_2 (1 μg/kg/day)	<0.05	
OVX VS. OVX+PM2.5 (2.5 mg/kg/day)	<0.001	
OVX VS. OVX+PM25 (25 mg/kg/day)	<0.001	
**Group comparison Training test (Day 5)**	**ANOVA followed by Tukey's post hoc test**	
	P	F ($DF_{between\ group}$, $DF_{residual}$)
Sham VS. OVX Vehicle–treated	<0.05	F(4,53) =8.562
OVX VS. OVX+E_2 (1 μg/kg/day)	<0.05	
OVX VS. OVX+PM2.5 (2.5 mg/kg/day)	<0.001	
OVX VS. OVX+PM25 (25 mg/kg/day)	<0.001	
**Group comparison Probe test**	**ANOVA followed by Tukey's post hoc test**	
	P	F ($DF_{between\ group}$, $DF_{residual}$)
Sham VS. OVX Vehicle–treated	<0.05	F(4,50)=4.627
OVX VS. OVX+E_2 (1 μg/kg/day)	<0.05	

OVX VS. OVX+PM2.5 (2.5 mg/kg/day)	Not significant	
OVX VS. OVX+PM25 (25 mg/kg/day)	<0.05	

2. Statistical analysis of Changes in uterus weight and volume and serum 17β—estradiol levels after the PM treatment

Table S4. One-way analysis of variance (ANOVA) test of the uterus weight and volume and serum 17β—estradiol levels after the PM treatment

**Group comparison (Uterus weight)**	**ANOVA followed by Tukey's post hoc test**	
	P	*F* ($DF_{between\ group}$, $DF_{residual}$)
Sham VS. OVX Vehicle-treated	<0.05	Do not test
OVX VS. OVX+E_2 (1 μg/kg/day)	<0.05	
OVX VS. OVX+PM2.5 (2.5 mg/kg/day)	<0.05	
OVX VS. OVX+PM25 (25 mg/kg/day)	<0.05	
**Group comparison (Uterus volume)**	**ANOVA followed by Tukey's post hoc test**	
	P	*F* ($DF_{between\ group}$, $DF_{residual}$)
Sham VS. OVX Vehicle-treated	<0.05	Do not test
OVX VS. OVX+E_2 (1 μg/kg/day)	<0.05	
OVX VS. OVX+PM2.5 (2.5 mg/kg/day)	<0.05	
OVX VS. OVX+PM25 (25 mg/kg/day)	<0.05	
**Group comparison (Serum E2)**	**ANOVA followed by Tukey's post hoc test**	
	P	*F* ($DF_{between\ group}$, $DF_{residual}$)
Sham VS. OVX Vehicle-treated	<0.05	$F(4,52)=10.218$
OVX VS. OVX+E_2 (1 μg/kg/day)	<0.001	
OVX VS. OVX+PM2.5 (2.5 mg/kg/day)	Not significant	
OVX VS. OVX+PM25 (25 mg/kg/day)	<0.05	

3. Statistical analysis of Effects of PM extract and estrogen on oxidative damage and antioxidant enzyme activities in the hippocampus and serum of OVX mice

Table S5. One-way analysis of variance (ANOVA) test of oxidative stress

**Group comparison (Hippocampus)**	**ANOVA followed by Tukey's post hoc test**	
	P	*F* ($DF_{between\ group}$, $DF_{residual}$)
Sham VS. OVX Vehicle-treated	<0.001	$F(3,18)=10.532$
OVX VS. OVX+E_2 (1 μg/kg/day)	<0.05	
OVX VS. OVX+PM2.5 (2.5 mg/kg/day)	Not significant	
OVX VS. OVX+PM25 (25 mg/kg/day)	<0.05	
**Group comparison (Serum)**	**ANOVA followed by Tukey's post hoc test**	
	P	*F* ($DF_{between\ group}$, $DF_{residual}$)
Sham VS. OVX Vehicle-treated	<0.001	$F(3,18)=7.351$
OVX VS. OVX+E_2 (1 μg/kg/day)	<0.05	

OVX VS. OVX+PM2.5 (2.5 mg/kg/day)	Not significant	
OVX VS. OVX+PM25 (25 mg/kg/day)	<0.05	

Table S6. One-way analysis of variance (ANOVA) test of SOD

Group comparison (Hippocampus)	ANOVA followed by Tukey's post hoc test	
	P	F ($DF_{between\ group}$, $DF_{residual}$)
Sham VS. OVX Vehicle-treated	<0.05	F(4,15) =15.189
OVX VS. OVX+E2 (1 μg/kg/day)	<0.001	
OVX VS. OVX+PM2.5 (2.5 mg/kg/day)	Not significant	
OVX VS. OVX+PM25 (25 mg/kg/day)	<0.05	
OVX+PM2.5 VS. OVX+PM25	<0.05	
**Group comparison (Serum)**	**ANOVA followed by Tukey's post hoc test**	
	P	F ($DF_{between\ group}$, $DF_{residual}$)
Sham VS. OVX Vehicle-treated	<0.05	F(4,25) =8.995
OVX VS. OVX+E2 (1 μg/kg/day)	<0.05	
OVX VS. OVX+PM2.5 (2.5 mg/kg/day)	Not significant	
OVX VS. OVX+PM25 (25 mg/kg/day)	<0.001	
OVX+PM2.5 VS. OVX+PM25	<0.001	

Table S7. One-way analysis of variance (ANOVA) test of CAT

Group comparison (Hippocampus)	ANOVA followed by Tukey's post hoc test	
	P	F ($DF_{between\ group}$, $DF_{residual}$)
Sham VS. OVX Vehicle-treated	<0.001	F(3,20) =10.024
OVX VS. OVX+E2 (1 μg/kg/day)	<0.001	
OVX VS. OVX+PM2.5 (2.5 mg/kg/day)	Not significant	
OVX VS. OVX+PM25 (25 mg/kg/day)	<0.001	
OVX+PM2.5 VS. OVX+PM25	<0.05	
**Group comparison (Serum)**	**ANOVA followed by Tukey's post hoc test**	
	P	F ($DF_{between\ group}$, $DF_{residual}$)
Sham VS. OVX Vehicle-treated	<0.05	F(3,22)) =12.244
OVX VS. OVX+E2 (1 μg/kg/day)	<0.001	
OVX VS. OVX+PM2.5 (2.5 mg/kg/day)	<0.05	
OVX VS. OVX+PM25 (25 mg/kg/day)	<0.001	
OVX+PM2.5 VS. OVX+PM25	<0.05	

3. Statistical analysis of the effects of PM extract and estrogen on proinflammatory cytokines and estrogen–mediated gene in hippocampus

Table S8. One–way analysis of variance (ANOVA) test of IL–1β mRNA

Group comparison	ANOVA followed by Tukey's post hoc test	
	P	*F* ($DF_{between\ group}$, $DF_{residual}$)
Sham VS. OVX Vehicle–treated	<0.05	$F(3,16)=6.023$
OVX VS. OVX+E_2 (1 μg/kg/day)	<0.05	
OVX VS. OVX+PM2.5 (2.5 mg/kg/day)	Not significant	
OVX VS. OVX+PM25 (25 mg/kg/day)	<0.05	

Table S9. One–way analysis of variance (ANOVA) test of IL–6 mRNA

Group comparison	ANOVA followed by Tukey's post hoc test	
	P	*F* ($DF_{between\ group}$, $DF_{residual}$)
Sham VS. OVX Vehicle–treated	<0.05	$F(3,18)=6.603$
OVX VS. OVX+E_2 (1 μg/kg/day)	<0.05	
OVX VS. OVX+PM2.5 (2.5 mg/kg/day)	Not significant	
OVX VS. OVX+PM25 (25 mg/kg/day)	<0.05	

Table S10. One–way analysis of variance (ANOVA) test of TNF–α mRNA

Group comparison	ANOVA followed by Tukey's post hoc test	
	P	*F* ($DF_{between\ group}$, $DF_{residual}$)
Sham VS. OVX Vehicle–treated	<0.05	$F(3,17)=6.635$
OVX VS. OVX+E_2 (1 μg/kg/day)	<0.05	
OVX VS. OVX+PM2.5 (2.5 mg/kg/day)	<0.05	
OVX VS. OVX+PM25 (25 mg/kg/day)	<0.05	

Table S11. One–way analysis of variance (ANOVA) test of PI3K mRNA

Group comparison	ANOVA followed by Tukey's post hoc test	
	P	*F* ($DF_{between\ group}$, $DF_{residual}$)
Sham VS. OVX Vehicle–treated	<0.05	$F(3,16)=14.034$
OVX VS. OVX+E_2 (1 μg/kg/day)	<0.05	
OVX VS. OVX+PM2.5 (2.5 mg/kg/day)	Not significant	
OVX VS. OVX+PM25 (25 mg/kg/day)	<0.05	

4. The HPLC chromatogram of partial purification of P. candollei var. mirifica extract

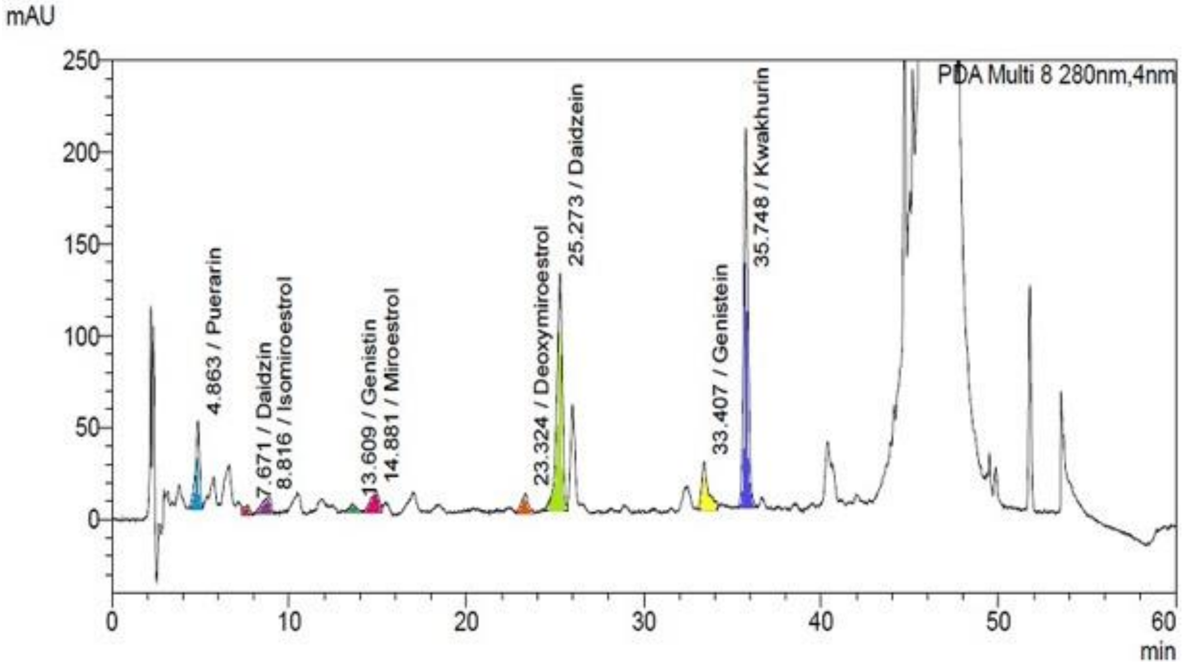

Figure S1 The HPLC chromatogram of partial purification of *P. candollei* var. mirifica extract shows in absorbance and retention time of phytoestrogen contents. The total retention times of nine phytoestrogens i.e. puerarin, daidzin, isomiroestrol, genistin, miroestrol, deoxymiroestrol, daidzein, genistein, and kwakurin are presented, respectively.
